# Supplementary material for: Ploidy and neuron size impact nervous system development and function in Xenopus
Source: Cell Rep. Author manuscript; Available in PMC 2026 Apr 29. (PMC13126290; doi:10.1016/j.celrep.2026.116969)
Supplement: 1 [file NIHMS2151772-supplement-1.pdf]

**Cell Reports, Volume *45***

## Supplemental information

# Ploidy and neuron size impact nervous system development and function in *Xenopus*

**Xiao Liu, Christine Wan, Sara Aijaz Shah, and Rebecca Heald**

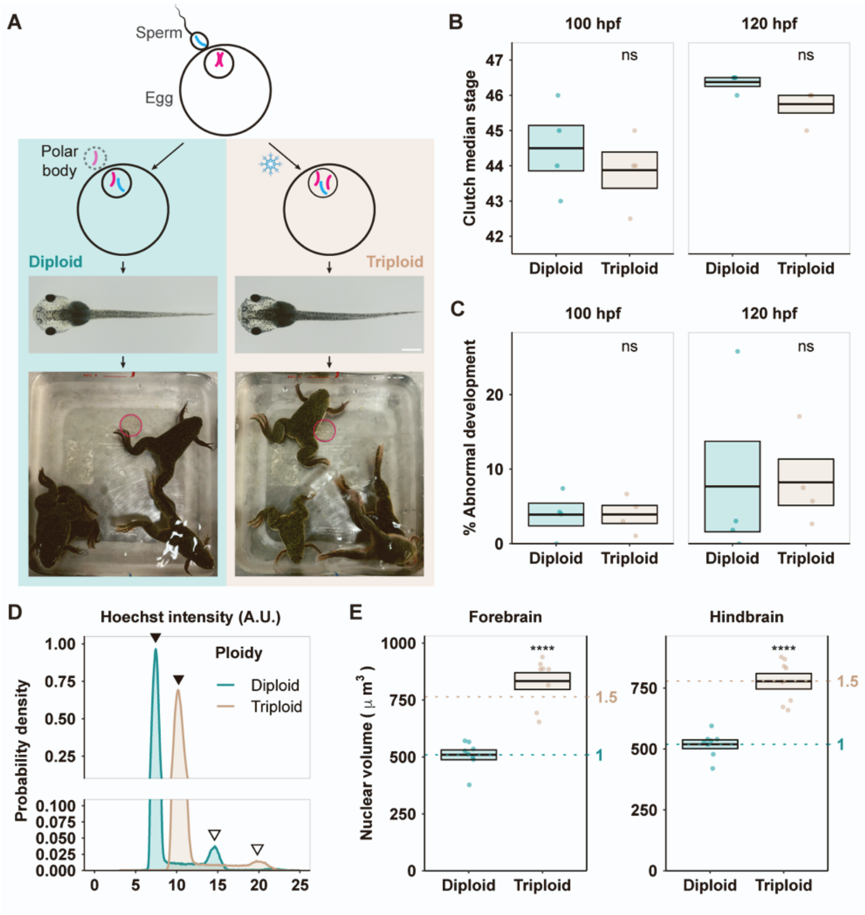


**Figure S1. Polyploid *Xenopus* as a model to study brain development**
Related to Figure 1.

(A) Top, diagram showing *Xenopus in vitro* fertilization without or with cold shock treatment, resulting in diploid or triploid embryos, respectively[S1]. Bottom, representative photos of stage 46 tadpoles (scale bar, 1 mm) and adult frogs (red dashed circle, a U.S. quarter as a size reference) of the indicated ploidy. Tadpoles and frogs shown are clutch mates. Frogs were raised by Clotilde Cadart[S2].

(B–C) Developmental stage (B) and percentage of embryos with gross abnormalities (C) of diploid and triploid clutch mates at the indicated hours post-fertilization (hpf). Each dot represents the median of one clutch. >300 tadpoles per ploidy per time point from 4 independent clutches were examined. Abnormal-looking tadpoles were not staged. Crossbars denote mean ± SEM. ns, not significant, paired Wilcoxon signed-rank test in B and paired t test in C.

(D) Representative density distribution of Hoechst intensity in diploid and triploid brain cells. Samples of both ploidies were obtained from the same clutch and Hoechst intensity of individual cells was analyzed by flow cytometry. Solid arrowheads denote $G_0/G_1$ peaks and open arrowheads denote $G_2$/M peaks.

(E) Nuclear volume of diploid and triploid neurons in the forebrain and hindbrain. Each dot represents the mean value of nuclei measured in one brain. 8 brains per ploidy across 3 independent clutches were examined. Crossbars indicate mean ± SEM and dotted lines mark 1- and 1.5-fold of diploid mean. ***, $p<0.001$; ****, $p<0.0001$, t test.

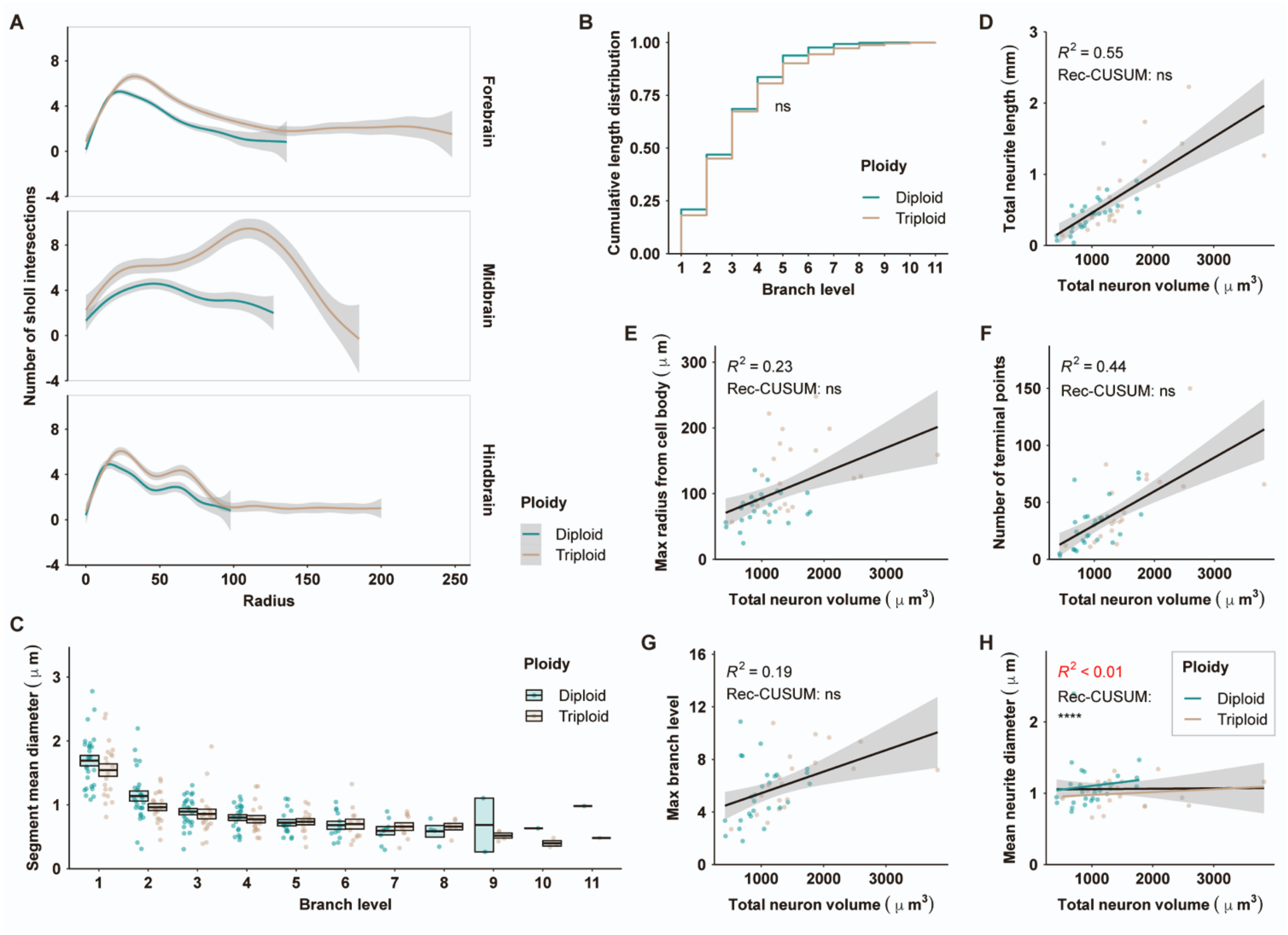


**Figure S2. Size and shape analysis of diploid and triploid neurons**

Related to Figure 1.

(A) Sholl analyses of diploid and triploid neurons in the indicated brain regions. The same neurons were used as in Figures 2C. Data were smoothed with a generalized additive model (GAM) and were presented as mean ± 95% confidence interval.

(B) Cumulative length distribution of neurites at different branch levels. ns, not significant, Kolmogorov-Smirnov test.

(C) Mean neurite segment diameter at different branch levels. Same data as in Figure 1I.

(D–H) Scatter plots of various size parameters versus total neuron volume: total neurite length (D), max radius from the cell body (E), number of terminal points (F), max branch level (G), and mean neurite diameter (H). Diploid and triploid data were combined, smoothed with a linear model, and presented as mean ± 95% confidence interval. Recursive CUSUM test[S3] was used to detect differences in regression coefficients between the diploid and triploid datasets. ****, $p<0.0001$; ns, not significant. In H, diploid and triploid regressions are shown separately due to the detection of a significant structural break in the combined model.

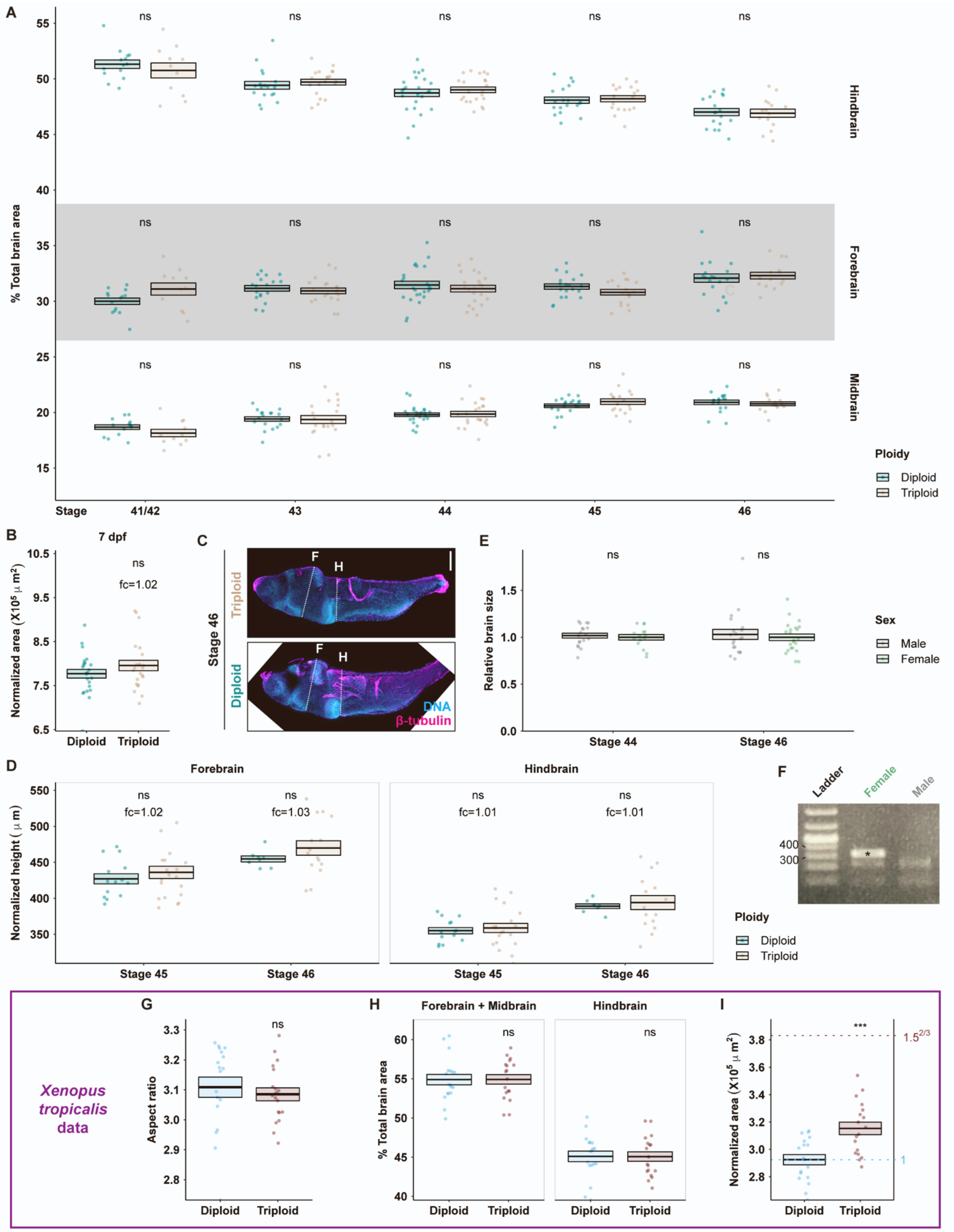

A
% Total brain area
Hindbrain
Forebrain
Midbrain
ns
Stage
41/42
43
44
45
46
Ploidy
Diploid
Triploid
B
7 dpf
Normalized area (X10⁵ μ m²)
fc=1.02
Diploid
Triploid
C
Stage 46
Triploid
Diploid
F
H
DNA
β-tubulin
E
Relative brain size
Stage 44
Stage 46
Sex
Male
Female
D
Forebrain
Hindbrain
Normalized height (μ m)
fc=1.02
fc=1.03
fc=1.01
Stage 45
Stage 46
F
Ladder
Female
Male
400
300
G
Xenopus tropicalis data
Aspect ratio
H
Forebrain + Midbrain
% Total brain area
I
Normalized area (X10⁵ μ m²)
***
1.5^2/3
1

**Figure S3. Triploid brains are morphologically similar to diploid brains**
Related to Figure 3.

(A) Comparison of the proportion of the forebrain, midbrain, and hindbrain in diploid and triploid brains across multiple developmental stages. Numbers of diploid/triploid brains examined were 15/11 at stage 41/42, 18/20 at stage 43, 24/22 at stage 44, 19/18 at stage 45, and 18/15 at stage 46. Brains were from 3 independent clutches.

(B) Brain area comparison of diploid and triploid tadpoles at 7 dpf (late stage 46 to stage 47). A total of 24 diploid and 23 triploid brains from 3 independent clutches were examined.

(C) Representative z-projected micrographs of side-mounted stage 46 diploid and triploid brains. Images were stitched from overlapping tiles. Dotted lines mark where the height measurements in E were taken. F, forebrain; H, hindbrain. Scale bar, 200 μm.

(D) Height comparison of diploid and triploid brains at two developmental stages. Numbers of diploid/triploid brains examined were 14/20 at stage 45 and 8/15 at stage 46. Brains were from 3 independent clutches. Heights were normalized to adjust for clutch variance.

(E) Relative size of female and male diploid brains. Numbers of male/female brains examined were 15/18 at stage 44 and 22/20 at stage 46. Brains were from 3 independent clutches. Brain size was divided by its clutch female mean to normalize against clutch variance.

(F) An example DNA gel used to determine the sex of tadpoles. The presence of a W chromosome-specific amplicon (315 bp, asterisk) indicates a female[S4].

(G–I) Comparisons of the aspect ratio (G), proportion of different brain regions (H), and normalized area (I) of diploid and triploid *X. tropicalis* brains at stage 46. 18 brains per ploidy from 3 independent clutches were examined.

In all box plots, each dot represents one brain. Crossbars denote mean ± SEM. *, $p<0.05$; **, $p<0.01$; ***, $p<0.001$; ****, $p<0.0001$; ns, not significant, t test.

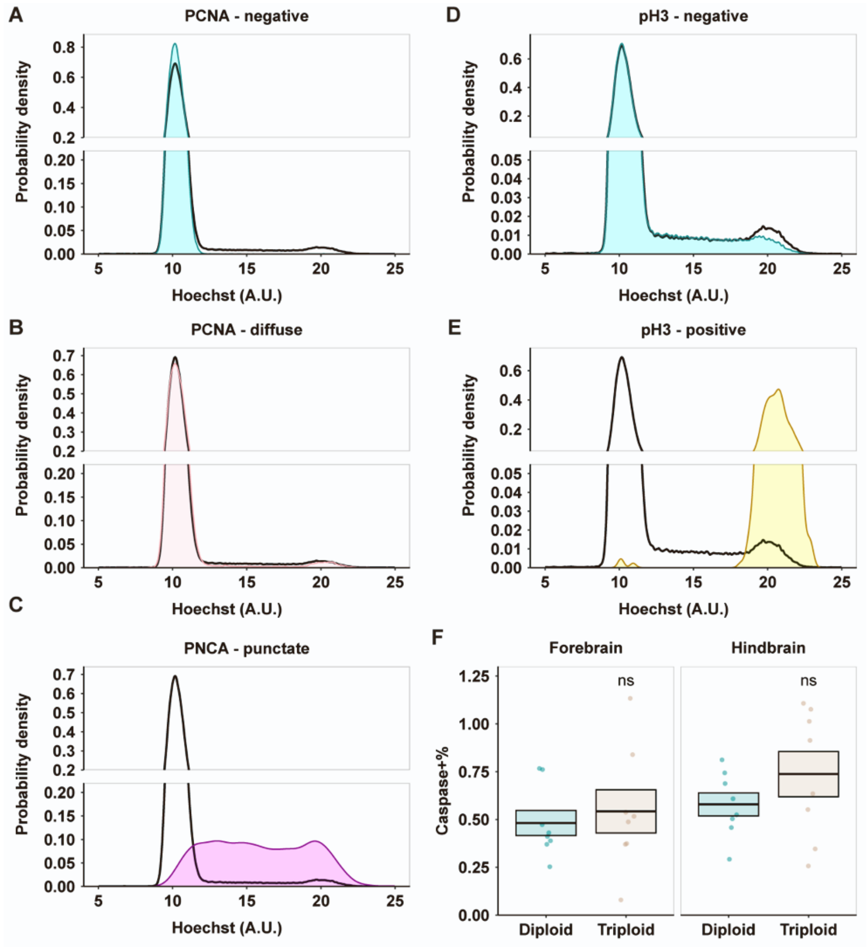


**Figure S4. Triploid brains show distinct cell birth/death dynamics**
Related to Figure 4.

(A–E) Probability density distributions of Hoechst intensity of the three different PCNA populations (A–C) and the two different pH3 populations (D–E) in a representative flow cytometry sample of dissociated brain cells at developmental stage 46. Colored curves show the distribution of the indicated population and black curves show the distribution of the total population as a reference.

(F) Ratio of cell death (marked by the positive staining of cleaved caspase-3[S5]) in the indicated brain region in diploid and triploid brains. Number of caspase-positive cells was normalized to total cell count in the same region. Each dot represents one brain. 8 brains per ploidy across 3 independent clutches were examined. Crossbars denote mean ± SEM. ns, not significant, t test.

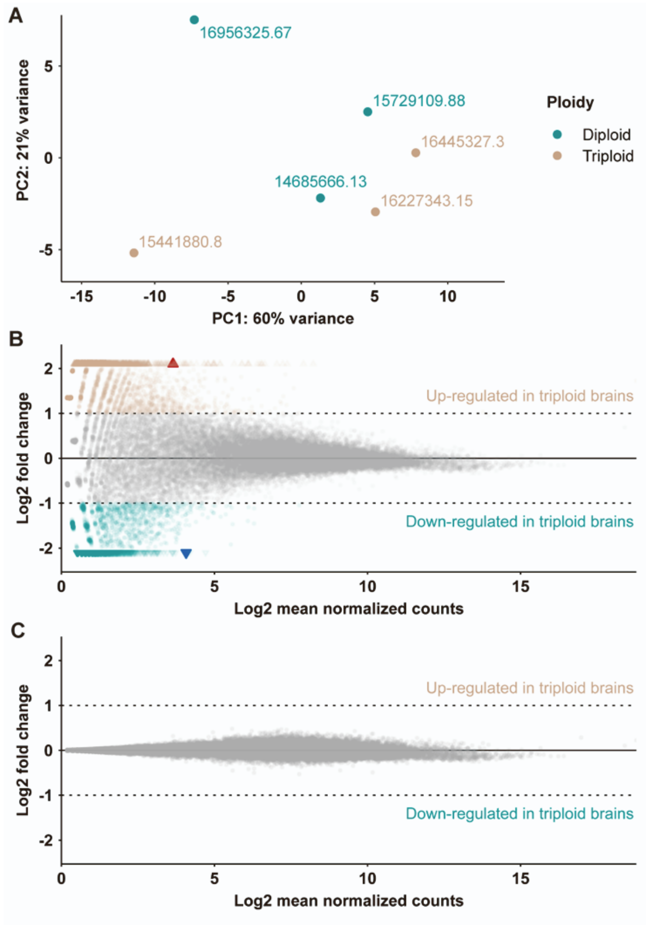


**Figure S5. Triploid and diploid brains possess similar transcriptional profiles**
Related to Figure 4.

(A) Principal component analysis (PCA) for brain-specific RNA-seq replicates. 3 replicates per ploidy were used and each replicate was prepared from >10 live-dissected brains of clutch-controlled, anesthetized stage 44–46 tadpoles. Total normalized count is shown for each replicate.

(B) Mean-Average (MA) plot showing log2 fold changes (triploid/diploid) of gene expression in tadpole brains (normalized by distribution). Points that fall outside of the y-axis limits are plotted as triangles. The two enlarged, colored triangles are the only two with adjusted p<0.1 and are both uncharacterized genes. Red, LOC121394545, predicted to encode E3 ubiquitin-protein ligase DCST1-like; blue, LOC121394266, predicted to encode general transcription factor II-I repeat domain-containing protein 2-like (Xenbase *Xenopus laevis* J strain 10.1).

(C) MA plot with log2 fold changes in B moderated with normal shrinkage to remove noise[S6]. No point falls outside of the y-axis limits. No point has adjusted p<0.1.

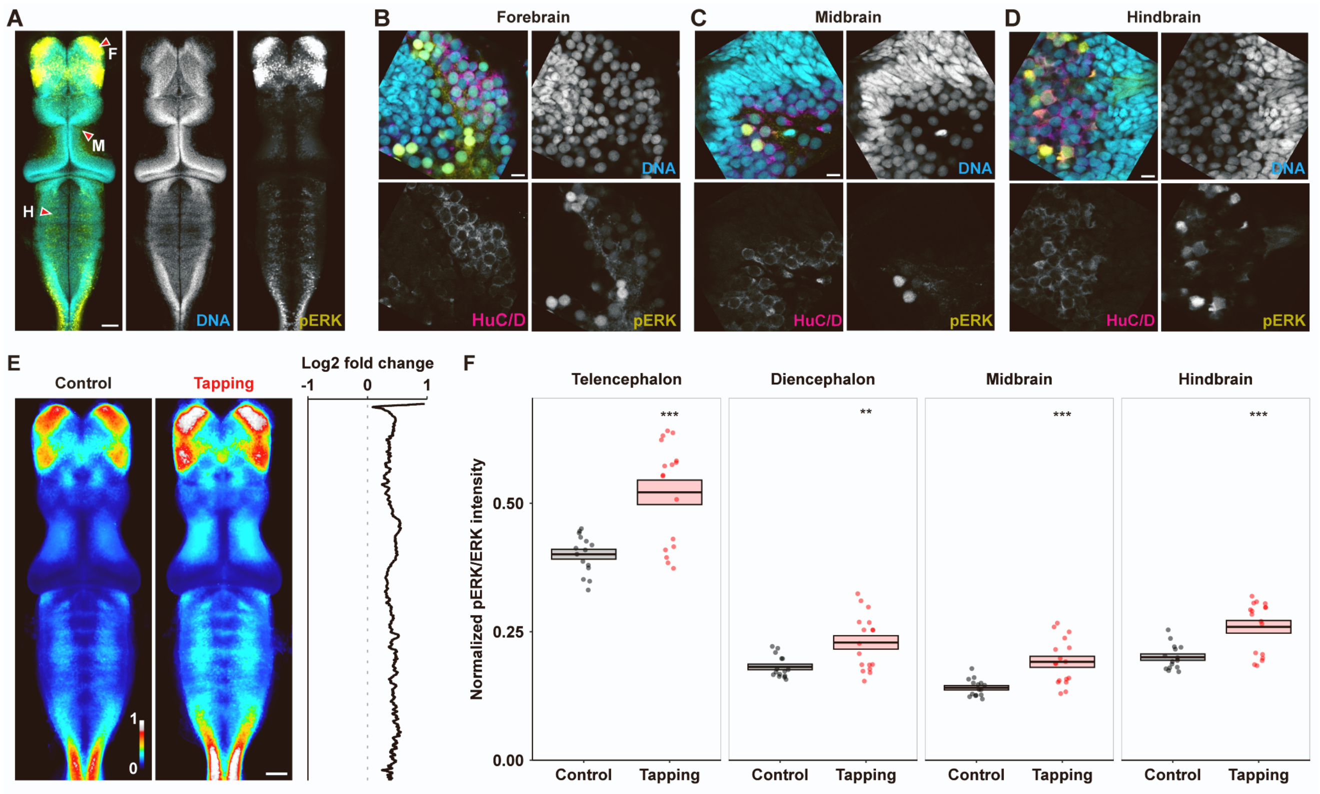


**Figure S6. pERK expression is neuronal and reflects neural activity**
Related to Figure 5.

(A) The reference brain (stage 46, diploid) used for image registration during pERK/ERK intensity measurements. Images shown were z-projected and stitched from overlapping tiles. Arrowheads mark locations where zoomed-in micrographs were taken in B–D. F, forebrain; M, midbrain; H, hindbrain. Scale bar, 100 µm.

(B–D). Representative micrographs of pERK and HuC/D co-staining in the forebrain (B), midbrain (C), and hindbrain (D) of stage 46 tadpoles. Images were rotated to match the orientation of whole-brain images in A. Scale bar, 10 µm.

(E) Heatmap showing normalized, averaged pERK/ERK intensity of 15 control brains and 17 brains of tadpoles after 15 min of dish tapping. Stage 46 diploid tadpoles from 3 independent clutches were used. Log2 fold changes (tapping/control) of pERK/ERK intensity along Y axis are plotted to the right. Scale bar, 100 µm.

(F) Comparison of pERK/ERK intensity in the indicated brain regions. Each dot represents one brain. Crossbars denote mean ±SEM. **, p<0.01; ***, p<0.001, t test.

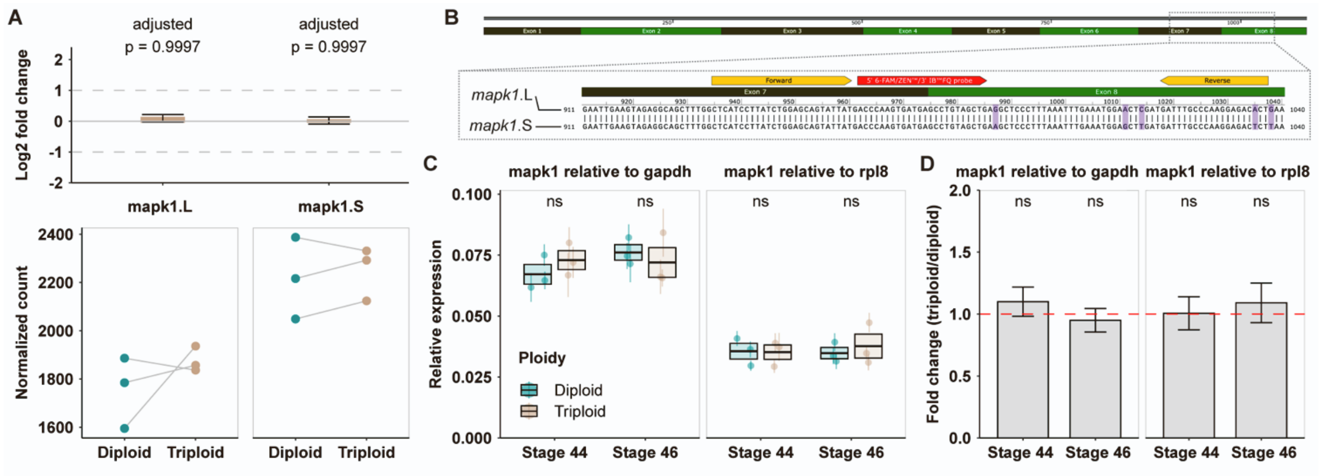


**Figure S7. ERK transcription is similar in diploid and triploid brains**
Related to Figure 5.

(A) Normalized counts and log2 fold changes (triploid/diploid) of the two ERK genes in *X. laevis* (*mapk1*, L and S homeologs). Grey lines connect samples from the same clutch. Data is from the same brain-specific RNA-seq as in Figures S5.

(B) Assay design for *mapk1* RT-PCR in C–D. Primers and exon-spanning probes amplify and detect both L and S homeologs. Mismatches in *mapk1*.L and *mapk1*.S sequences are highlighted in purple.

(C) Relative mapk1 expression compared to housekeeping genes *gapdh* and *rpl8* in diploid and triploid tadpole brains at two developmental stages. Crossbars denote mean ±SEM of 3 biological replicates, and point ranges denote mean ±SEM of 3 technical replicates of that biological replicate. ns, not significant, paired t test.

(D) Fold changes (triploids/diploids) of relative *mapk1* expression. Error bars denote mean ±SEM of 3 biological replicates. Red dotted line marks a fold change of 1 (no change). ns, not significant, one sample t test against a mean of 1.

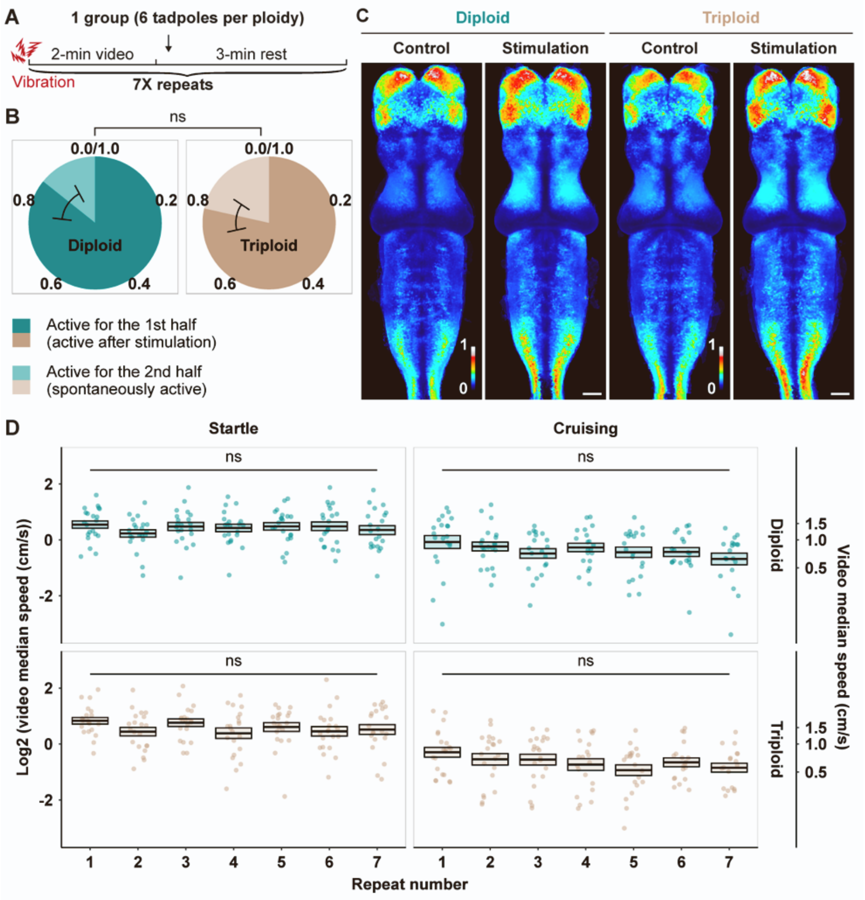


**Figure S8. Using the swimming assay to assess stimulated brain activity and tadpole behavior**
Related to Figure 6.

(A) Diagram showing the time scheme of the swimming assay.

(B) Break-down of the "half active" category in Figure 6B. Error bars mark 95% confidence interval. ns, not significant, Fisher's exact test.

(C) Heatmap showing normalized, averaged pERK/ERK intensity in brains of control and stimulated tadpoles. Numbers of brains averaged were 8, 8, 8, and 7, from left to right. Tadpoles from 2 independent clutches were stimulated with program-controlled vibration on 7 dpf and processed immediately after stimulation. Scale bar, 100 μm.

(D) Swimming speeds of tadpoles as they experienced repeated stimulation. Each dot represents the geometric mean of all TRex-tracked speeds in one video. Data from 4–7 dpf were pooled for this analysis. Crossbars denote mean ± SEM. ns, not significant, ANOVA test.

| Day post fertilization (dpf) | 3 | 4 | 5 | 6 | 7 |
|---|---|---|---|---|---|
| Clutches | 1 | 3 | 3 | 3 | 3 |
| Groups per clutch | 1 | 1–2 | 2 | 2 | 2 |
| Total videos | 7 | 28 | 42 | 42 | 42 |
| Scored manually | ✓ | ✓ | ✓ | ✓ | ✓ |
| Trex tracked | | ✓ | ✓ | ✓ | ✓ |

**Table S1. Number of replicates used for the swimming assay**
Related to Figure 6.

| **Fold change** (p-value) | **Diploid vs. Triploid** | | **Control vs. Stimulation** | |
|---|---|---|---|---|
| **Brain region** | **Control** | **Stimulation** | **Diploid** | **Triploid** |
| Telencephalon | **1.11** (0.252) | **0.95** (0.523) | **1.33** (0.002) | **1.14** (0.202) |
| Diencephalon | **1.20** (0.142) | **1.03** (0.852) | **1.29** (0.030) | **1.10** (0.509) |
| Midbrain | **1.25** (0.094) | **1.01** (0.823) | **1.31** (0.016) | **1.06** (0.305) |
| Hindbrain | **1.22** (0.082) | **1.03** (0.926) | **1.35** (0.025) | **1.14** (0.631) |

**Table S2. Detailed statistical analysis for data shown in Figure 6E**
Related to Figure 6.

For each comparison (t test), fold change is shown in bold with p-value in parentheses.

## SUPPLEMENTAL REFERENCES

[S1] Gibeaux, R., and Heald, R. (2019). Generation of Xenopus Haploid, Triploid, and Hybrid Embryos. Methods Mol. Biol. Clifton NJ *1920*, 303–315. https://doi.org/10.1007/978-1-4939-9009-2_18.

[S2] Cadart, C., Bartz, J., Oaks, G., Liu, M.Z., and Heald, R. (2023). Polyploidy in *Xenopus* lowers metabolic rate by decreasing total cell surface area. Curr. Biol. *33*, 1744-1752.e7. https://doi.org/10.1016/j.cub.2023.03.071.

[S3] Brown, R.L., Durbin, J., and Evans, J.M. (1975). Techniques for Testing the Constancy of Regression Relationships over Time. J. R. Stat. Soc. Ser. B Methodol. *37*, 149–192.

[S4] Mawaribuchi, S., Takahashi, S., Wada, M., Uno, Y., Matsuda, Y., Kondo, M., Fukui, A., Takamatsu, N., Taira, M., and Ito, M. (2017). Sex chromosome differentiation and the W- and Z-specific loci in *Xenopus laevis*. Dev. Biol. *426*, 393–400. https://doi.org/10.1016/j.ydbio.2016.06.015.

[S5] Crowley, L.C., and Waterhouse, N.J. (2016). Detecting Cleaved Caspase-3 in Apoptotic Cells by Flow Cytometry. Cold Spring Harb. Protoc. *2016*. https://doi.org/10.1101/pdb.prot087312.

[S6] Love, M.I., Huber, W., and Anders, S. (2014). Moderated estimation of fold change and dispersion for RNA-seq data with DESeq2. Genome Biol. *15*, 550. https://doi.org/10.1186/s13059-014-0550-8.
